# Molecular Characterization of the 2020 Outbreak of Lumpy Skin Disease in Nepal

**DOI:** 10.3390/microorganisms10030539

**Published:** 2022-02-28

**Authors:** Pragya Koirala, Irene Kasindi Meki, Manju Maharjan, Bharani Kumar Settypalli, Salina Manandhar, Sanjay Kumar Yadav, Giovanni Cattoli, Charles Euloge Lamien

**Affiliations:** 1 Central Veterinary Laboratory, Veterinary Complex, Tripureshwor, Kathmandu 44600, Nepal; paggya2000@gmail.com (P.K.); meet_davet@hotmail.com (M.M.); smanandhar76@yahoo.com (S.M.); 2 Animal Production and Health Laboratory, Joint FAO/IAEA Division of Nuclear Techniques in Food and Agriculture, Department of Nuclear Sciences and Applications, International Atomic Energy Agency, Wagramer Strasse 5, P.O. Box 100, A-1400 Vienna, Austria; t.b.k.settypalli@iaea.org (B.K.S.); g.cattoli@iaea.org (G.C.); c.lamien@iaea.org (C.E.L.); 3 Veterinary Laboratory, Biratnagar 56613, Nepal; sanjayyadav160@hotmail.com

**Keywords:** LSDV, Nepal, sequence, outbreak

## Abstract

Lumpy skin disease (LSD) is a transboundary viral disease of cattle and buffaloes transmitted by blood-feeding vectors and causes high morbidity and low-to-moderate mortality. Since the first observation of LSD in Zambia in 1929, it has spread in cattle populations across African countries, the Middle East, Europe, and Asia. Following the recent outbreaks of LSD in South Asian countries such as India and Bangladesh, the disease was first reported in cattle farms in Nepal in June 2020. This study investigated the Nepalese LSD outbreak and confirmed that the disease spread rapidly to three neighboring districts in a month, infecting 1300 animals. Both cattle and buffaloes showed common clinical signs of LSD, with the exception that the buffaloes presented small nodular lesions without centered ulcerations. The collected samples were first tested for the presence of LSDV by real-time PCR. We further applied molecular tools, RPO30, GPCR, EEV glycoprotein gene, and B22R, for additional characterization of the LSDV isolates circulating in Nepal. Using a PCR-based Snapback assay, we confirmed that samples collected from cattle and buffaloes were positive of LSDV. Furthermore, sequence analysis (phylogenetic and multiple sequence alignments) of four selected LSDV genes revealed that the Nepal LSDVs resemble the Bangladesh and Indian isolates and the historic isolates from Kenya. We also highlight the importance of a unique B22R gene region harboring single-nucleotide insertions in LSDV Neethling and LSDV KSGPO-240 vaccine strains, enabling us to differentiate them from the Nepalese isolates and other fields isolates. This study demonstrates the importance of disease surveillance and the need to determine the source of the disease introduction, the extent of spread, modes of transmission, and the necessary control measures.

## 1. Introduction

Lumpy skin disease (LSD) is a transboundary viral disease of cattle and buffaloes with a significant economic impact on the livestock industry, including financial losses [1,2]. The etiological agent is the LSD virus (LSDV), a DNA virus of the genus Capripoxvirus (CaPV) in the Poxviridae family. Sheeppox virus (SPPV) and goatpox virus (GTPV) are two other capripoxviruses that infect sheep and goats, respectively. They are known to be antigenically similar to LSDV [3].

LSDV is transmitted by blood-feeding vectors such as stable flies, mosquitoes, and ticks [4,5,6]. LSD causes high morbidity and low-to-moderate mortality. The disease may vary from subclinical infection to death based on the virus strain, vector prevalence, age, or the host’s immune status [7]. The clinically sick cattle present anorexia, enlarged superficial lymph nodes, nasal discharge, watery eyes, and firm flat-topped papules and nodules all over the body, including head, neck, udder, scrotum, and buccal mucosa [8,9].

LSD was first observed in Zambia in 1929, then spread rapidly in cattle population across African countries and became endemic. From 2012, the disease reached the Middle East, Europe, and South Asia [10] successively. While the initial LSDV genome in Africa was stable or had minor differences for decades, including for vaccines related field isolates which were only sequenced recently [11], the field isolates recovered after the transcontinental spread of LSD revealed significant genomic variabilities [12,13]. For instance, the molecular characterization of the field LSDV isolates from Russia and China showed vaccine-like profiles attributable to the recombination events between the Neethling vaccine strain and field isolates related to LSDV KSGPO-240 [14,15,16]. Notably, the recombinant LSDVs appeared more pathogenic than the typical field isolates [16,17].

Additionally, capripoxviruses are known to be cross-reactive. Thus, SPPV or GTPV based vaccines have also been used against LSDV [3,18]. Therefore, several molecular-based techniques were developed to correctly identify species within the Capripoxvirus genus and further differentiate live attenuated vaccines from field isolates and the recombinant LSDV strains [19,20,21,22,23,24,25]. Those assays target selected viral genomic regions, such as the G-protein-coupled receptor (GPCR), the RNA polymerase 30 kDa subunit (RPO30), the CaPV homolog of the variola virus B22R gene, and the EEV glycoprotein [26,27,28,29,30].

Nepal reported its first LSD case in cattle farms in June 2020 [31], following the recent outbreaks of LSD in South Asian countries such as China, India, and Bangladesh [32,33,34]. These first cases in Nepal occurred in a district near the Indian border. By the end of July 2020, the disease had spread to three more neighboring districts in Nepal, affecting 1300 animals. Samples collected from the suspected cases were confirmed to be LSD positive at the Central Veterinary Laboratory (CVL), Veterinary Complex, Kathmandu, Nepal, by Real-time polymerase chain reaction (RT-PCR) of the GPCR gene [31]. The current study aims to confirm the occurrence of LSDV in cattle and buffaloes in Nepal and provide a molecular characterization of the field LSDV isolates circulating in Nepal.

## 2. Materials and Methods

### 2.1. Study Area and Sample Collection

**Figure 1:**
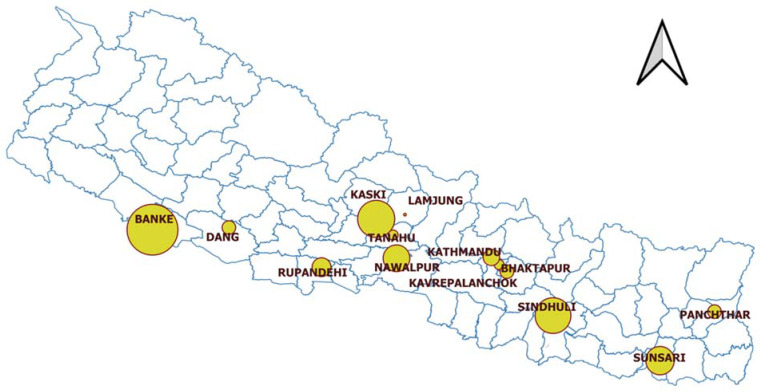
Map showing the geographical locations and sample size of the LSD-affected districts in Nepal between August and October 2020.

This study includes the samples collected following the spread of LSD after the first outbreak of the disease in Nepal. During the episode, suspected cases of LSD were noticed in cattle and captive buffaloes as of June 2020 in the Eastern part of Nepal. The suspected animals were sick and showed LSD-like symptoms, including high fever and nodular lesions on different body parts. Later, other provinces reported similar symptoms on cattle and buffaloes. The Central Veterinary Laboratory confirmed LSD, enabling the notification to OIE of the first LSD case in Nepal in July 2020. The disease then spread progressively throughout the country. For further investigation of the outbreak, samples from different tissues (scab, serum, nasal swab, ocular swab, and skin nodules) of the clinically infected cattle and buffaloes were collected from thirteen districts (representing four provinces) between August and October 2020 for further characterization (Figure 1). The total number of susceptible cattle and buffalo populations and the reported morbidities and mortalities in the thirteen districts are summarized in Table 1. The collected samples were transported to the Central Veterinary Laboratory (CVL).

### 2.2. Sample Processing and DNA Extraction

The samples (scab, nasal swab, skin nodules, serum) were processed in CVL. The skin nodules were cut into small pieces and ground in PBS. Scabs and nasal swabs were collected in PBS, centrifuged, and the supernatant was collected in sterile vials. 200 μL of the supernatant was used for DNA extraction using PureLinkTM Viral RNA/DNA Mini Kit (Invitrogen, Waltham, MA, USA) according to the manufacturer’s instructions. The DNA was eluted in 50 μL of elution buffer and stored at −80 °C until further use.

### 2.3. Molecular Detection and Differential Diagnosis

A previously developed Snapback assay was applied to detect and genotype the capripoxviruses found in the collected samples [29]. Table 2 presents the sequences of the primers used in this assay. This assay enables distinguishing the three genotypes of capripoxviruses: SPPV, GTPV, or LSDV based on the differences in the melting temperature (Tm) of the snapback stem and the PCR amplicons obtained following the fluorescence melting curve analysis. The PCR mixture was set up in a 20 μL reaction volume, containing 10 μL of the iQsupermix (Bio-Rad), 500 nM of the Snapback forward primer, 40 nM of the reverse primer, and 2 μL of template DNA. The PCR program consisted of an initial denaturation step at 95 °C for 3 min, followed by 45 cycles at 95 °C for 15 s and 58 °C for 80 s with the fluorescence reading at the end, followed by 95 °C for 1 min, 40 °C for 1 min, and melting between 40–85 °C at 10 s/0.5 °C with the fluorescence reading at each °C then 37 °C for 1 min.

### 2.4. Amplification of Selected Genes and Sequencing

To confirm the identity and further characterize the LSDV positive samples, four genes: the RPO30, GPCR, Extracellular enveloped virus (EEV) glycoprotein, and CaPV homolog of the variola virus B22R genes were amplified by PCR as previously described using the primers listed in Table 2. All PCRs were performed in a 20 μL reaction volume containing 500 nM of each of the forward and the reverse primers, 200 uM of dNTPs, 1x PCR buffer (Qiagen), 1.25 U of Taq DNA polymerase (Qiagen), and 2 μL template DNA. After amplification, the PCR products were analyzed on a 2% gel electrophoresis and visualized on a Gel Documentation System (Bio-Rad). The PCR products were purified using the PCR clean-up system kit (Promega), following the manufacturer’s instructions. The products were sequenced using both forward and reverse primers at LGC Genomics (Germany). The nucleotide sequences were deposited in the GenBank database under the accession numbers OL689584 to OL689619.

### 2.5. Sequence and Phylogenetic Analysis

The raw sequences were assembled using the Vector NTI software (Invitrogen) version 11.5. The sequences for each targeted gene for all the samples, together with representative CaPV sequences retrieved from GenBank, were aligned with MEGA7 using the Muscle algorithm and the codon option [35]. The alignment files of the complete RPO30 and GPCR gene sequences were converted to a Nexus format using Seaview. The Bayesian phylogenetic inference was performed using BEAST v1.8.4 [36]. The log files generated by BEAST were inspected using the TRACER v1.7.1 program to determine the optimum burn-in value number, based on the effective sample sizes (ESS). TreeAnnotator was then used to generate the maximum clade credibility (MCC) file based on the burn-in value. The trees were visualized and annotated using the Interactive tree of life (iTOL) [37]. The LSDV clusters based on the presence/absence of insertion in the GPCR or EEV glycoprotein were visualized with the RPO30 tree. The multiple sequence alignments of the partial EEV glycoprotein and B22R genes were visualized using BioEdit (v7.2.6).

### 2.6. Targeted Nanopore Sequencing of Short PCR Fragments in the Buffalo Serum

Following the unsuccessful amplification and sequencing of the four targeted genes in the positive buffalo samples, we have undertaken to amplify and sequence short genome fragments to confirm the presence of the LSDV genome. Using the fluorescence resonance energy transfer assay (FRET) [31], and Snapback assay [29] primers, in the absence of probes or ds DNA intercalating dyes, we have amplified a short segment of each the RPO30 gene (96 bp) and GPCR gene (200 bp). The resulting amplicons were purified and sequenced using the MinION device of nanopore as described below: The purified PCR products were used to prepare the library using Amplicons by Ligation Kit (SQK-LSK109) according to Oxford Nanopore Technologies (ONT) standard protocols. The prepared library was sequenced on R9 flow cell. Data acquisition and real-time basecalling was performed with the MinKNOW v3.5.10.1 and the integrated Guppy v3.2.6 (ONT).

The raw sequences were cleaned to remove the adaptors, using porechop (v 0.2.4), and the low-quality and short reads, using NanoFilt (v2.8.0). The quality of the remaining reads was assessed with NanoStat (v1.6.0). After mapping the cleaned raw reads against the GPCR gene and the RPO30 gene of LSDV NI-2490 (NC_003027), using minimap2 (v2.24), SAMtools (v1.11) was used to generate Mpileup files and performed variant calling using BCFtools (v1.9). The sorted reads were displayed within the Integrated Genome Viewer (IGV, v2.8.0) browser for visualization. The consensus sequences were produced with vcfutils.pl (VCFtools v0.1.16) and seqtk (v1.3.106) and compared to the corresponding fragments in publicly available LSDVs sequences by multiple sequence alignments using BioEdit (v7.2.6).

## 3. Results

### 3.1. Clinical Signs and Symptoms and Outbreak Investigations

**Figure 2:**
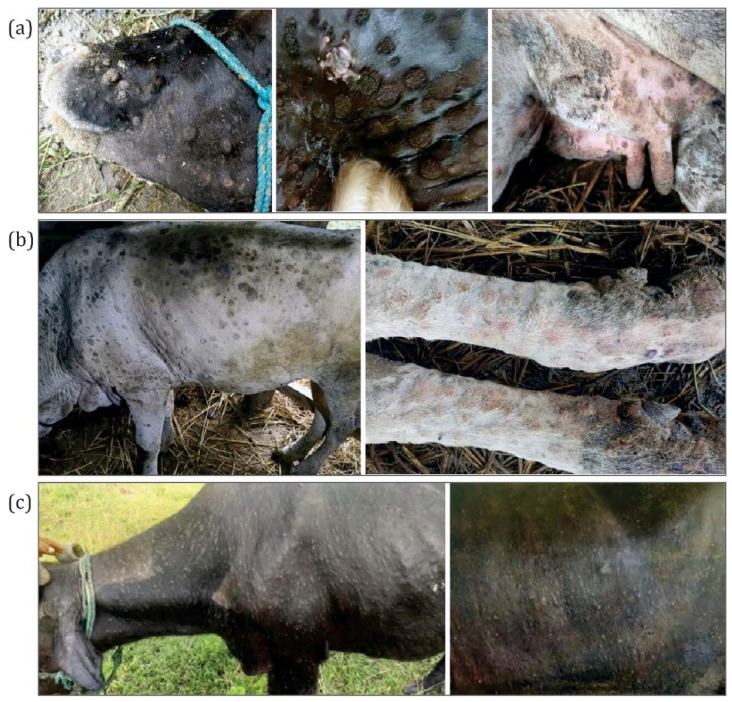
Lesions in LSD suspected animals. (**a**) Local Terai cow with nodular skin lesions over the face, neck, and udder. (**b**) Local cow with nodular skin lesions all over the body and limbs. (**c**) Buffalo showing small skin nodules all over the body.

Most of the affected cattle and buffaloes showed common clinical signs and symptoms such as fever (40–41.5 °C), loss of appetite, nasal and ocular discharge, and generalized skin nodules all over the body. Besides, the sick animals presented enlarged sub-scapular and pre-femoral lymph nodes and a sharp drop in milk production. Some infected cattle also presented necrotic plaques in the oral and nasal mucous membranes, ulcerated nodules, and scabs. However, in the case of buffaloes, only small nodular lesions without centered ulcerations were observed. Figure 2 illustrates the nodular skin lesions over the face, neck, limbs, and all over the body of cattle and buffalo.

Ninety-two cattle and nine buffaloes out of 30,868 and 9397 susceptible cattle and buffaloes were clinically affected from the thirteen districts. The overall morbidity rate was 0.3% for cattle and 0.1% for buffaloes. The morbidity rate in different districts ranged from 0.05 to 1.7% for cattle and 0.08 to 0.3% for buffaloes (Table 1). No deaths were reported in the districts included in this study. The vectors known for transmitting LSDV, such as ticks and mosquitoes, were also observed within the farms.

### 3.2. Molecular Detection and Diagnosis of LSDV

**Figure 3:**
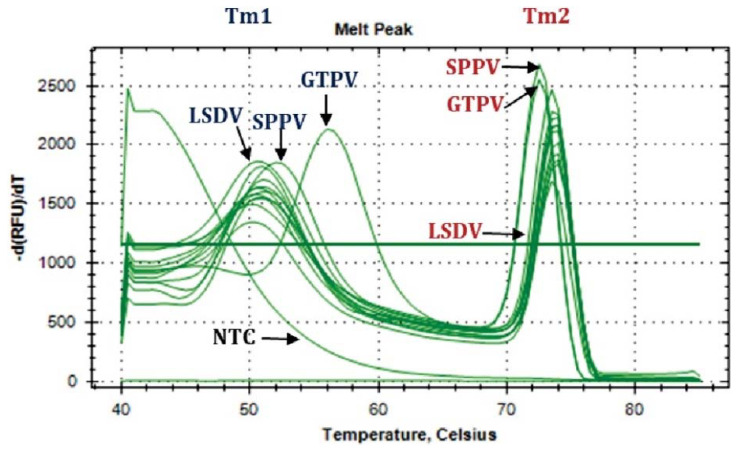
Melting peaks (Tm) of samples from Nepal compared to the positive control of GTPV, SPPV, and LSDV. Both melting temperatures for the snapback stem (Tm1) and the PCR amplicons (Tm2) are shown.

As summarized in Table 3, CaPV DNA was detected in 14 out of 20 samples (12 cattle and two buffalo samples) using the PCR-based Snapback assay. Furthermore, based on the melting temperature of the Snapback stem (Tm1) and that of the PCR amplicons (Tm2), the CaPV positive samples were confirmed to be LSDV positive, as shown by the corresponding melting temperatures (Figure 3 and Table 3). Subsequently, only samples with a Cq of 30.00 or less were sequenced for further analyses.

### 3.3. Amplification and Sequencing

Nine LSDV positive cattle samples were successfully amplified and sequenced for the complete RPO30 and GPCR genes and partial EEV glycoprotein and B22R genes. After the quality check and editing, the appropriate size, 606, 1146, 327, and 832 bp, for the RPO30, GPCR, EEV, and B22R genes, respectively, the sequences were deposited to GenBank. In addition, for one bufallo sample, we have successfully amplified and sequenced using nanopore technology two short fragments in the RPO30 and GPCR genes, corresponding to the target of the the snapback and FRET assays (Figure S1).

### 3.4. Sequence and Phylogenetic Analysis

**Figure 4:**
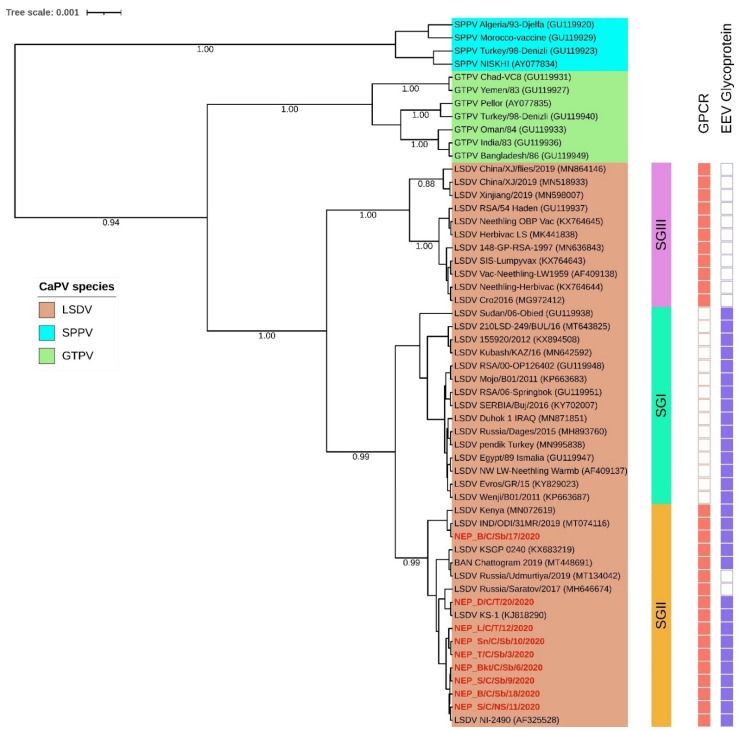
Maximum clade credibility (MCC) tree based on the complete RPO30 gene sequences of CaPVs with LSDVs from Nepal (in red) visualized on iTOL, together with isolates clustering based on the presence (filled box) or absence (empty box) of sequence insertion in the GPCR and the EEV glycoprotein genes.

The multiple sequence alignments showed 100% similarity among the nine Nepalese LSDV positive cattle samples for all the four targeted genes. Besides, sequence alignment of the short segments of the RPO30 and GPCR sequences of the LSDV positive buffalo sample, with other Nepalese LSDVs, showed 100% similarity to the LSDV isolate in cattle (Figure S2). In the phylogenetic analysis based on the complete RPO30 gene sequences, Nepal isolates clustered in the LSDV subgroup SG II. SG II also included recent LSDV isolates from Bangladesh and India (2019), two historical LSDVs, LSDV Kenya and LSDV NI-2490. In addition, the vaccine strain LSDV KSGPO-240 and two recombinant LSDVs from Russia (LSDV Udmurtiya/2019, and LSDV Saratov/2017) also belonged to SG II (Figure 4). The multiple sequence alignment of the complete GPCR gene sequences showed a 12-nucleotide insertion in Nepal isolates similar to the recombinant LSDVs from Russia, LSDVs from Bangladesh and India, LSDV NI-2490, LSDV Kenya, and Chinese LSDVs. This marker is typically present in LSDV vaccine strains such as LSDV KSGPO-240 and LSDV Neethling vaccine strain as well as LSDVs in SG II and SG III (Figure 4). However, in the GPCR phylogenetic analysis, the Nepalese isolates clustered in sub-group SG I with only LSDVs from Bangladesh and India, and LSDV KSGPO-240, LSDV NI-2490, and LSDV Kenya and not with the recombinant LSDVs from Russia (Figure S3).

**Figure 5:**
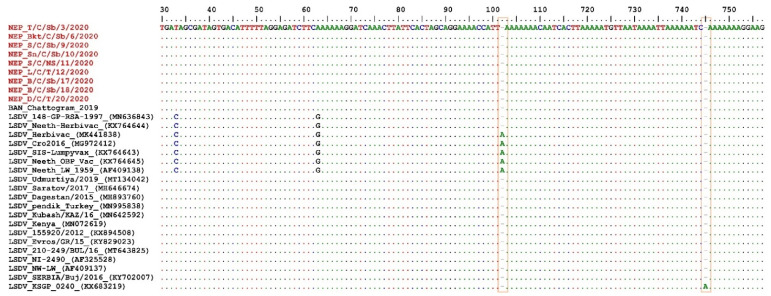
Multiple sequence alignments of the partial nucleotide sequences of the B22R gene. The Nepal isolates (in red) were aligned with representative LSDV sequences retrieved from GenBank. The nucleotide insertion in LSDV Neethling and LSDV KSGPO-240 vaccines that are absent in Nepal isolates are shown in the blocks. The dots indicate the identical nucleotides in the alignment.

The multiple sequence alignment of the EEV glycoprotein gene showed a 27-nucleotide insertion (175-201) in Nepalese LSDVs, similar to LSDVs from Bangladesh, LSDV KSGPO-240, LSDV NI-2490, and LSDV Kenya. Interestingly, this 27-nucleotide insertion differentiated SGI and SGII from SGIII, which include LSDV Neethling derived vaccines and Chinese LSDVs, and from the recombinant LSDVs from Russia (Figure 4 and Figure S4). Moreover, the multiple sequence alignment of the targeted B22R fragment reiterated that the Nepalese isolates were like the LSDV NI-2490 and LSDV Kenya and LSDVs from Bangladesh. However, they differed from LSDV Neethling and LSDV KSGPO-240 derived vaccines with nucleotide insertion at nucleotide alignment positions 102 and 745, respectively (Figure 5).

## 4. Discussion

Although LSDV has spread from Africa to the Middle East and East Europe since 2012, the disease only reached East and South Asia in 2019, affecting China, India, Bangladesh, Bhutan, Vietnam, Thailand, Hong Kong, and Myanmar [38,39]. The first suspected LSD cases in Nepal were reported in June 2020 in the Morang district near the Indian border and were confirmed by molecular techniques [31]. The disease spread within four weeks to three neighboring districts, with a series of the suspected diseased animals showing typical LSD clinical signs such as generalized nodular skin disease, sharp drop in milk production, and infertility [8]. The overall morbidity rate observed in this study was low, and no mortality cases were reported. Low mortality rate to a total absence of mortality was also reported in recent LSD outbreaks in India (0%), Bangladesh (0.002%), Russia (0%), and Greece (0.40%) [32,34,40,41]. The main explanation for the low to the absence of mortality could be the low virulence of this LSDV strain or the influence of host factors such as the species, breed, age, immune status, and stage of production of the host [7].

The Snapback assay confirmed that all the CaPV positive samples from cattle or buffaloes contained LSDV and not GTPV or SPPV [29]. Furthermore, the multiple sequence alignments of each of the four targeted LSDV genes showed that all Nepalese LSDVs were identical. Although we have failed to amplify and sequence the full length of the four targeted genes of the LSDV from buffalo, we successfully obtained short RPO30 and GPCR fragments by nanopore sequencing. The multiple sequence alignment of these short fragments confirmed the presence of the LSDV genome in the buffaloes and also suggested that the buffalo LSDV isolates could be similar to the cattle LSDV isolates in Nepal. It has been reported that LSDV viremia is relatively short-lived in blood, 4–11 days post-infection [42]. Therefore, it is possible that the LSDV DNA in buffalo blood samples was degraded, and only short fragments targeted by the LSDV Snapback and FRET primers could be amplified and not the long PCR fragments targeted for Sanger sequencing in this study. This agrees with Zeynalova et al., 2016 [43], who previously concluded that skin nodules were better-suited samples for LSDV PCR detection than blood samples.

In the RPO30 gene tree, the Nepalese LSDVs clustered within LSDV subgroup SG II, together with LSDV isolates from the neighboring Bangladesh and India, as well as LSDV Kenya and LSDV NI-2490, two historical LSDVs collected in Kenya before the 1960s, two recombinant LSDV field isolates from Russia and the LSDV KSGPO-240 derived vaccines [14,32]. However, the Nepalese isolates clustered separately from LSDVs from neighboring China located within LSDV subgroup SG III. In addition, the GPCR sequences of the Nepalese LSDVs presented a 12-nucleotide insertion like LSDV NI-2490, LSDV Kenya, LSDVs from Bangladesh, India, and China, LSDV vaccines, and recombinant LSDVs from Russia. This insertion differentiates the Nepalese isolates from other GPCR LSDV subgroup SG I members from the Middle East, Europe, and Africa [27].

Furthermore, the 27-nucleotide deletion in the EEV glycoprotein gene in Nepalese LSDVs, similar to LSDVs from Bangladesh and LSDV NI-2490 and LSDV Kenya, two historical LSDVs from Kenya, distinguished them from the Chinese LSDV isolates and the recombinant LSDVs from Russia. Lastly, we have also exploited a unique region of the B22R gene to differentiate the Nepalese isolates from LSDV Neethling and LSDV KSGPO-240 vaccine, using single nucleotide insertions present in each of the two vaccine strains (positions 102 and 745, respectively) [28]. Therefore, based on sequence analysis for all the four selected LSDV genes: the RPO30, GPCR, EEV glycoprotein, and the B22R, the Nepal LSDV isolates resemble the Bangladesh isolates and the historic isolates NI-2490 and LSDV Kenya [32]. Although only the full-length RPO30 and GPCR gene sequences of the Indian isolate are available, both are 100% identical to the Nepal isolate, which suggests that they are very closely related [34]. Notably, the LSD outbreak in Nepal was reported soon after the LSD outbreak in the neighboring countries, Bangladesh and India. The fact that the strains circulating in these three countries are similar suggests that they may have a common source of infection for these countries. Although the Nepalese isolates were similar to the KSGPO-240 vaccine strain, there was no report of a vaccination program against LSD in Nepal before the outbreak. Previous studies have shown that using the KSGPO-240 vaccine could lead to lesions in vaccinated animals similar to the LSD symptoms [44,45]. However, since the disease is transmitted by arthropod vectors capable of moving across countries such as those reported in the farms in Nepal, it is likely that the LSD virus was introduced to Nepal by animal importation from neighboring countries or by mechanical transmission [4,5]. It was recently suggested that the introduction of LSD in Nepal in June 2020 was likely due to the continuous flow of informal cross-border movements of cattle from India to districts in eastern Nepal, usually by foot, given there are no official records of live cattle or buffalo imports from India in the fiscal year 2019 [46]. Currently, the Department of Livestock Services (DLS) has enforced LSD control measures such as quarantine at the border, control of vectors (flies, mosquitoes and ticks) that play an important role in LSDV transmission as well as continuous surveillance [31]. Regardless, the source of the strains currently circulating in South Asia with genetic features resembling “historic” field African strains and vaccine strains (KSGPO-240) remains obscure and deserves further investigation.

## 5. Conclusions

In conclusion, based on both clinical signs and molecular characterization, this study confirmed an outbreak of LSDV in both cattle and buffaloes in Nepal. In addition, the circulating Nepalese LSDV isolates were different from the common LSDV field isolates encountered in Africa, the Middle East, and Europe and the newly emerged LSDV variants in Russia and China. However, the Nepalese LSDV isolates were closely related to the historic LSDVs from Kenya and those circulating in the neighboring countries, Bangladesh and India. Therefore, following the current LSDV outbreak in Nepal and the neighboring countries successively, this study reiterates the importance of surveillance and early detection of diseases as a crucial control measure for disease outbreaks.

## Figures and Tables

**Table 1:** Features of the LSD outbreak in Nepal in cattle and buffaloes between August and October 2020.

District	Administration Region	No. of Susceptible Cases	No. of Clinically Affected Cases	No. of Dead Cases	Morbidity Rate (%)	Species	Type of Sample	Collection Date	Sample ID
Sunsari	Province I	3780	8	0	0.21	Cattle	Scab	October 2020	Sn/C/Sb/10/2020
Sunsari		728	2	0	0.27	Buffalo	Serum	October 2020	Sn/B/S/15/2020
Panchthar		238	3	0	1.26	Cattle	Serum	October 2020	P/C/S/7/2020
Kathmandu	Bagmati Province	809	4	0	0.49	Cattle	Nasal swab	August 2020	Kt/C/NS/4/2020
Kathmandu						Cattle	Nasal swab	August 2020	Kt/C/NS/5/2020
Bhaktapur		758	2	0	0.26	Cattle	Scab	October 2020	Bkt/C/Sb/6/2020
Sindhuli		1969	15	0	0.76	Cattle	Scab	September 2020	S/C/Sb/9/2020
Sindhuli						Cattle	Nasal swab	September 2020	S/C/NS/11/2020
Kavre		3987	3	0	0.075	Buffalo	Serum	September 2020	Kr/B/S/8/2020
Kaski	Gandaki Province	4682	4	0	0.08	Buffalo	Scab	August 2020	K/B/Sb/1/2020
Kaski						Buffalo	Scab	August 2020	K/B/Sb/2/2020
Kaski		3170	12	0	0.38	Cattle	Serum	September 2020	K/C/S/16/2020
Tanahu		4164	2	0	0.05	Cattle	Scab	August 2020	T/C/Sb/3/2020
Lamjung		1100	1	0	0.09	Cattle	Tissue	September 2020	L/C/T/12/2020
Nawalpur		1799	9	0	0.50	Cattle	Serum	September 2020	N/C/S/14/2020
Rupandehi	Lumbini Province	5500	5	0	0.09	Cattle	Ocular swab	September 2020	R/C/OS/13/2020
Banke		1623	28	0	1.72	Cattle	Scab swab	October 2020	B/C/Sb/17/2020
Banke						Cattle	Scab swab	October 2020	B/C/Sb/18/2020
Dang		5958	3	0	0.05	Cattle	Tissue (Skin nodules)	October 2020	D/C/T/19/2020
Dang						Cattle	Tissue (Skin nodules)	October 2020	D/C/T/20/2020
**Total**		30868	92	0	0.3	Cattle			
		9397	9	0	0.09	Buffalo			

**Table 2:** List of the primers used for Snapback assay and amplification and sequencing of the targeted genes. The snapback tail of 16 bases is shown as underlined.

Target Gene	Primer	Amplicon Size
RPO30 Snapback	Cp-HRM-SBF-5′-ggTGTAGTACGTATAAGATTATCGTATAGAAACAAGCCTTTA-3′	SNAP BACK assay
	Cp-HRM1R-5′-AATTTCTTTCTCTGTTCCATTTG-3′	
GPCR_ FRET	CpRt Forward- 5′-GATAGTATCGCTAAACAATGG-3′	200 bp
	CpRt Reverse- 5′-ATCCAAACCACCATACTAAG-3′	
RPO30	Cp-OL1F 5′-CAGCTGTTTGTTTACATTTGATTTTT-3′	554 bp
	Cp-OL1R 5′-TCGTATAGAAACAAGCCTTTAATAGA-3′	
	Cp-OL2F 5′-TTTGAACACATTTTATTCCAAAAAG-3′	520 bp
	Cp-OL2R 5′-AACCTACATGCATAAACAGAAGC-3′	
GPCR	CpGPCR-OL1F-5′-TGAAAAATTAATCCATTCTTCTAAACA-3′	684 bp
	CpGPCR-OL1R-5′-TCATGTATTTTATAACGATAATGCAAA-3′	
	CpGPCR-OL2F-5′-TTAGCGGTATAATCATTCCAAATA -3′	603 bp
	CpGPCR-OL2R-5′-GCGATGATTATGATGATTATGAAGTG-3‘	
	CpGPCR-OL3F-5′-CACAATTATATTTCCAAATAATCCAA -3′	617 bp
	CpGPCR-OL3R-5′-TGTACATGTGTAATTTTAATGTTCGTA-3′	
EEV glycoprotein gene (ORF LSDV126)	EEVGly F- 5′-ATGGGAATAGTATCTGTTGTATACG-3′	250 bp
	EEVGly R-5′-CGAACCCCTATTTACTTGAGAA-3′	
B22R	B22R F- 5′-TCATTTTCTTCTAGTTCCGACGA-3′	863 bp
	B22R R- 5′-TTCGTTGATGATAAATAACTGGAAA-3′	

**Table 3:** PCR-based snap back assay results for detection and genotyping of LSDV.

S. No.	Sample ID	Host	SNAP BACK Assay Analysis			Remarks
			Cq	Tm1 (°C)	Tm2 (°C)	
1	K/B/Sb/1/2020	Buffalo	N/A	N/A	N/A	Negative
2	K/B/Sb/2/2020	Buffalo	N/A	N/A	N/A	Negative
3	T/C/Sb/3/2020	Cattle	20.18	51.00	74.00	LSDV
4	Kt/C/NS/4/2020	Cattle	28.73	50.50	74.00	LSDV
5	Kt/C/NS/5/2020	Cattle	N/A	N/A	N/A	Negative
6	Bkt/C/Sb/6/2020	Cattle	24.25	51.00	74.00	LSDV
7	P/C/S/7/2020	Cattle	37.38	50.50	73.50	LSDV
8	Kr/B/S/8/2020	Buffalo	24.86	50.00	73.50	LSDV
9	S/C/Sb/9/2020	Cattle	22.48	51.00	74.00	LSDV
10	Sn/C/Sb/10/2020	Cattle	19.83	51.00	74.00	LSDV
11	S/C/NS/11/2020	Cattle	24.83	51.00	74.00	LSDV
12	L/C/T/12/2020	Cattle	25.14	51.00	74.00	LSDV
13	R/C/OS/13/2020	Cattle	N/A	N/A	N/A	Negative
14	N/C/S/14/2020	Cattle	N/A	N/A	N/A	Negative
15	Sn/B/S/15/2020	Buffalo	36.34	51.00	73.50	LSDV
16	K/C/S/16/2020	Cattle	N/A	N/A	N/A	Negative
17	B/C/Sb/17/2020	Cattle	24.97	51.00	74.00	LSDV
18	B/C/Sb/18/2020	Cattle	29.28	50.50	74.00	LSDV
19	D/C/T/19/2020	Cattle	31.02	50.00	73.50	LSDV
20	D/C/T/20/2020	Cattle	22.87	50.50	74.00	LSDV
GTPV	GTPV positive control	N/A	27.82	56.00	72.50	GTPV
SPPV	SPPV positive control	N/A	27.88	52.00	72.50	SPPV
LSDV	LSDV positive control	N/A	24.90	50.50	73.50	LSDV
NTC	RNAse free water	N/A	N/A	N/A	N/A	Negative

## Data Availability

The DNA sequences generated and used in the analysis for this study are available in the GenBank under the accession numbers OL689584 to OL689619.

## Supplementary Materials

The following are available online at https://www.mdpi.com/article/10.3390/microorganisms10030539/s1, Figure S1: Visualization of aligned nanopore sequencing reads for two targeted fragments in the LSDV positive Buffalo sample (Kr/B/S/8/2020) from Nepal. The reads were mapped against (a) RPO30 and (b) GPCR genes of the LSDV NI-2490 (NC_003027) and displayed in the IGV browser. Figure S2: Multiple sequence alignments of the partial nucleotide sequences of (a) RPO30 and (b) GPCR genes of the Nepal LSDV isolates from cattle (in red) and buffalo (in green) aligned with representative LSDV sequences retrieved from GenBank. The dots indicate the identical nucleotides in the alignment. Figure S3: Maximum clade credibility (MCC) tree based on the complete GPCR gene sequences of CaPVs, with LSDVs from Nepal (in red), visualized on iTOL. Figure S4: Multiple sequence alignments of the partial nucleotide sequences of the EEV glycoprotein gene. The Nepal isolates (in red) were aligned with representative LSDV sequences retrieved from GenBank. A 27-nucleotide deletion absent in Nepal isolates is highlighted in the box. The dots indicate the identical nucleotides in the alignment.
