# Supplementary material for: Molecular Characterization of the 2020 Outbreak of Lumpy Skin Disease in Nepal
Source: Microorganisms. 2022 Feb 28;10(3):539. doi: 10.3390/microorganisms10030539 (PMC8954389; doi:10.3390/microorganisms10030539)
Supplement: Supplementary file 1 [file microorganisms-10-00539-s001.zip › microorganisms-1601887-supplementary.pdf]

(a)

(b)

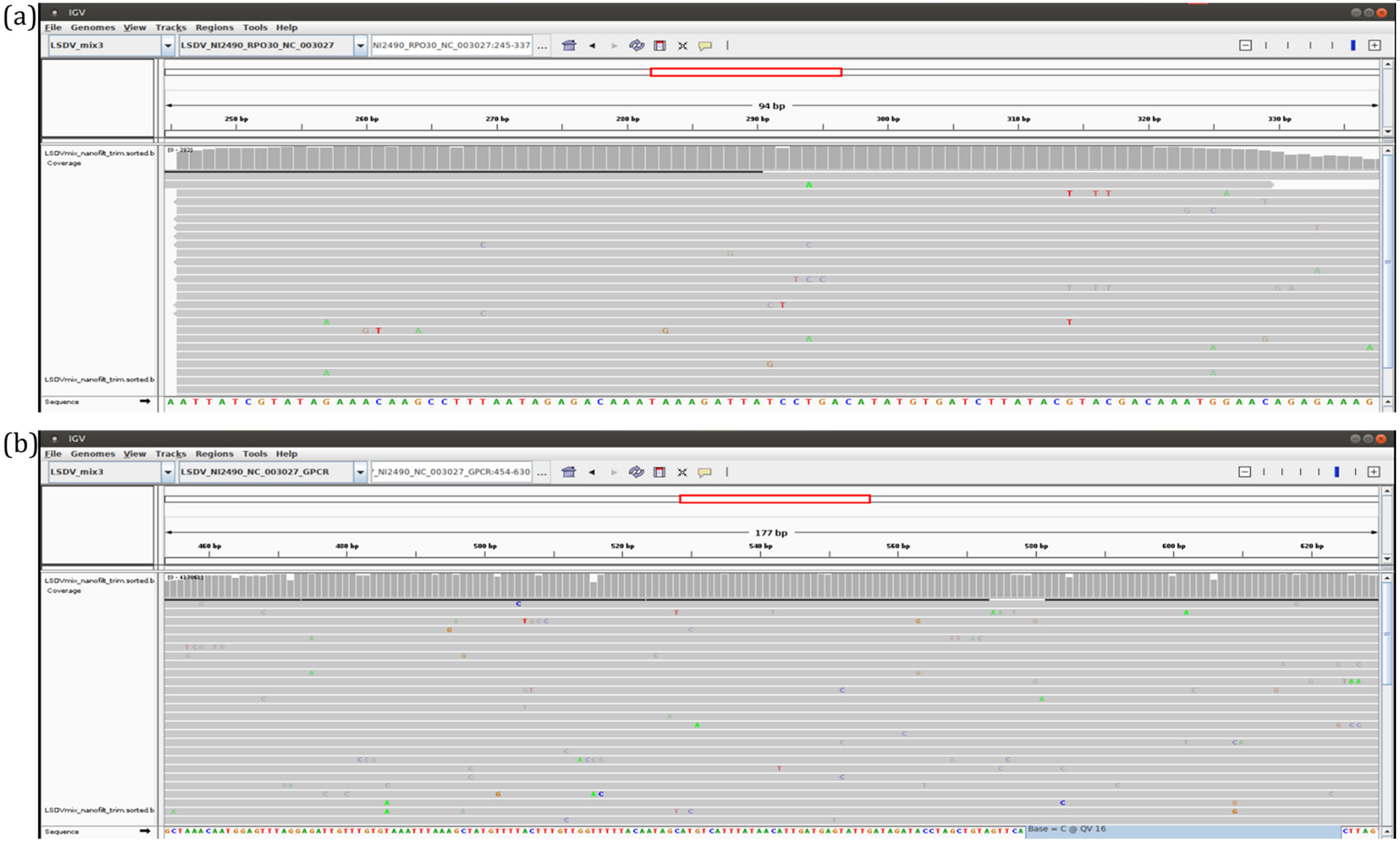

Figure S1. Visualization of aligned nanopore sequencing reads for two targeted fragments in the LSDV positive Buffalo sample (Kr/B/S/8/2020) from Nepal. The reads were mapped against (**a**) RPO30 and (**b**) GPCR genes of the LSDV NI-2490 (NC_003027) and displayed in the IGV browser.

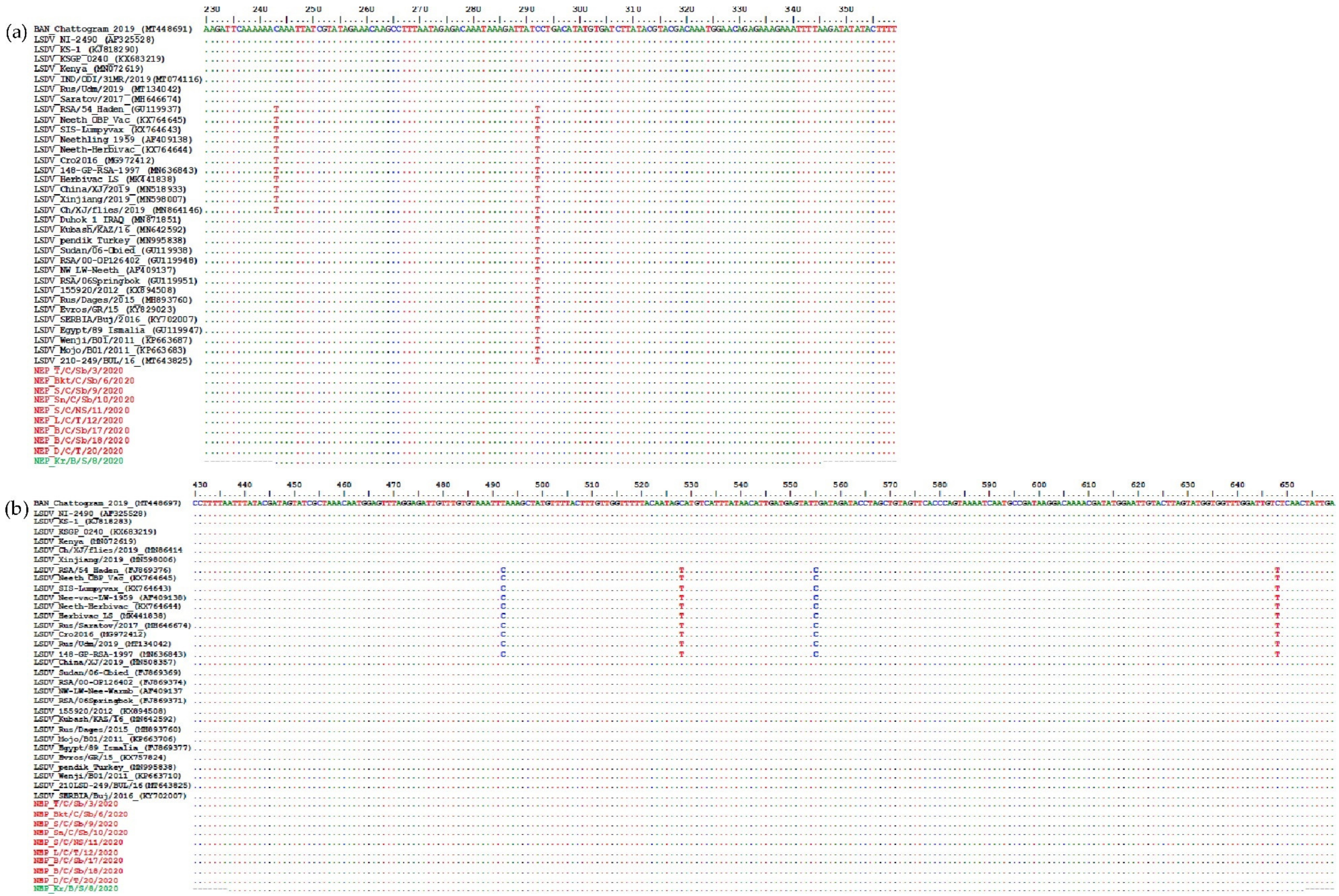

Figure S2. Multiple sequence alignments of the partial nucleotide sequences of (**a**) RPO30 and (**b**) GPCR genes of the Nepal LSDV isolates from cattle (in red) and Buffalo (in green) aligned with representative LSDV sequences retrieved from GenBank. The dots indicate the identical nucleotides in the alignment.

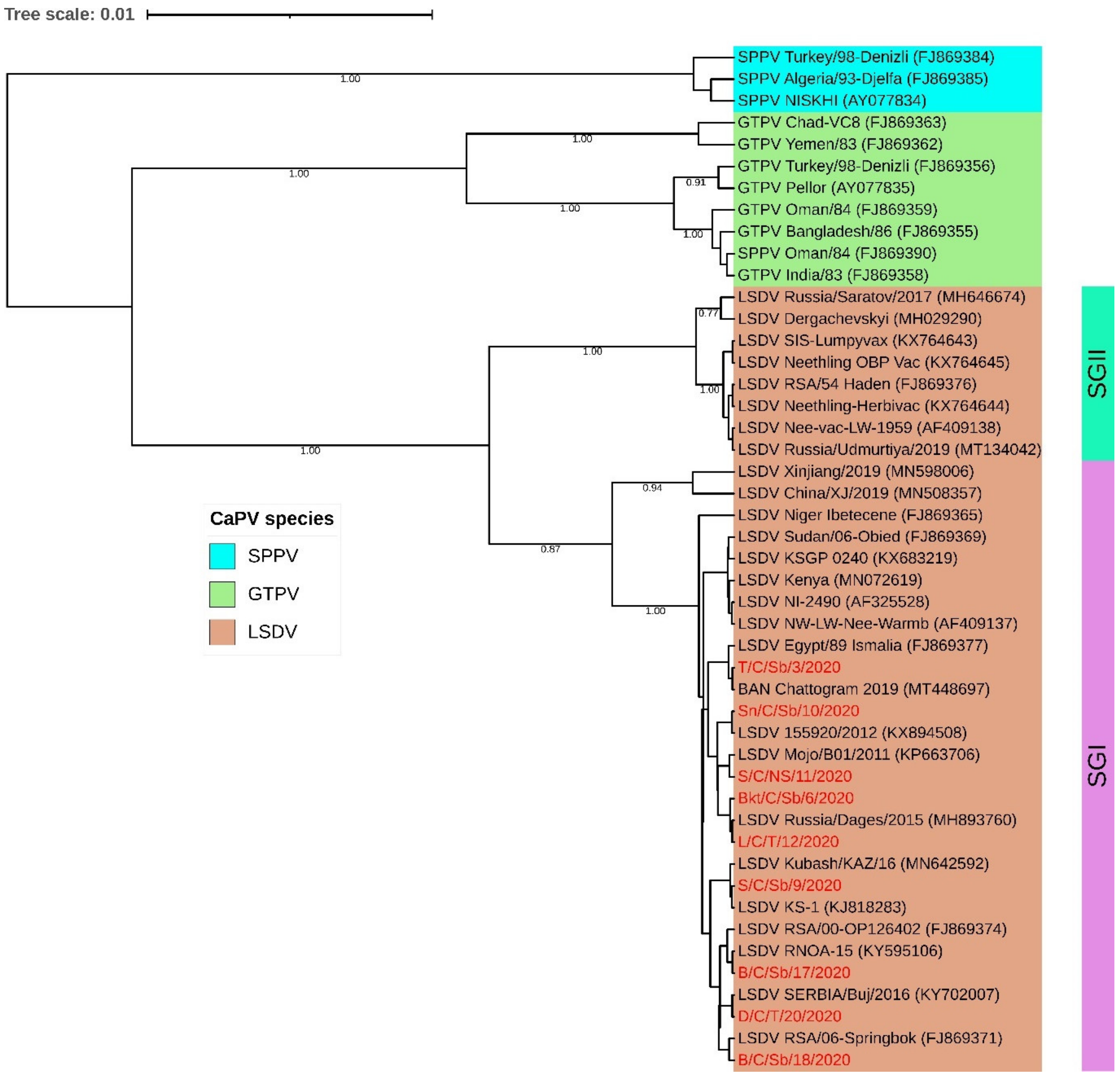

Figure S3. Maximum clade credibility (MCC) tree based on the complete GPCR gene sequences of CaPVs, with LSDVs from Nepal (in red), visualized on iTOL.

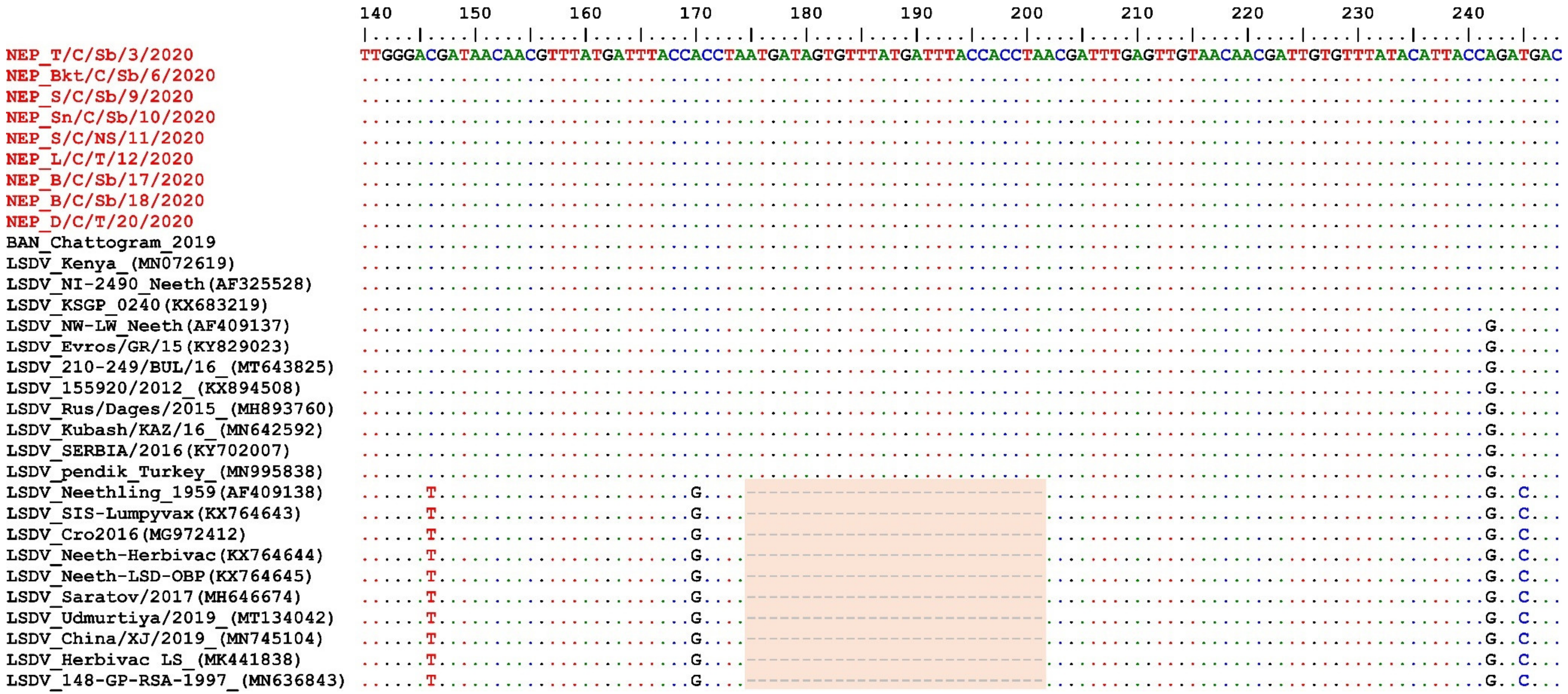

Figure S4. Multiple sequence alignments of the partial nucleotide sequences of the EEV glycoprotein gene. The Nepal isolates (in red) were aligned with representative LSDV sequences retrieved from GenBank. A 27-nucleotide deletion absent in Nepal isolates is highlighted in the box. The dots indicate the identical nucleotides in the alignment.
